# Genome-Wide Association Study Identifies *CDKN1A* as a Novel Locus Associated with Muscle Fiber Composition

**DOI:** 10.3390/cells11233910

**Published:** 2022-12-02

**Authors:** Ekaterina A. Semenova, Hirofumi Zempo, Eri Miyamoto-Mikami, Hiroshi Kumagai, Andrey K. Larin, Rinat I. Sultanov, Konstantin A. Babalyan, Andrey V. Zhelankin, Takuro Tobina, Keisuke Shiose, Ryo Kakigi, Takamasa Tsuzuki, Noriko Ichinoseki-Sekine, Hiroyuki Kobayashi, Hisashi Naito, Jatin Burniston, Edward V. Generozov, Noriyuki Fuku, Ildus I. Ahmetov

**Affiliations:** 1 Department of Molecular Biology and Genetics, Federal Research and Clinical Center of Physical-Chemical Medicine of Federal Medical Biological Agency, 119435 Moscow, Russia; 2 Research Institute of Physical Culture and Sport, Volga Region State University of Physical Culture, Sport and Tourism, 420138 Kazan, Russia; 3 Faculty of Health and Nutrition, Tokyo Seiei College, Tokyo 124-0025, Japan; 4 Graduate School of Health and Sports Science, Juntendo University, Chiba 270-1695, Japan; 5 Leonard Davis School of Gerontology, University of Southern California, Los Angeles, CA 90089, USA; 6 Faculty of Nursing and Nutrition, University of Nagasaki, Nagasaki 851-2195, Japan; 7 Faculty of Education, University of Miyazaki, Miyazaki 889-2192, Japan; 8 Faculty of Management & Information Science, Josai International University, Chiba 283-8555, Japan; 9 Faculty of Pharmacy, Meijo University, Nagoya 468-8503, Japan; 10 Faculty of Liberal Arts, The Open University of Japan, Chiba 261-8586, Japan; 11 Department of General Medicine, Mito Medical Center, Tsukuba University Hospital, Ibaraki 310-0015, Japan; 12 Research Institute for Sport and Exercise Sciences, Liverpool John Moores University, Liverpool L3 5AF, UK; 13 Department of Physical Education, Plekhanov Russian University of Economics, 115093 Moscow, Russia; 14 Laboratory of Genetics of Aging and Longevity, Kazan State Medical University, 420012 Kazan, Russia

**Keywords:** DNA, GWAS, genotype, genetic markers, muscle fibers, athletic performance, exercise, endurance, talent identification, sports, sprinters

## Abstract

Muscle fiber composition is associated with physical performance, with endurance athletes having a high proportion of slow-twitch muscle fibers compared to power athletes. Approximately 45% of muscle fiber composition is heritable, however, single nucleotide polymorphisms (SNP) underlying inter-individual differences in muscle fiber types remain largely unknown. Based on three whole genome SNP datasets, we have shown that the rs236448 A allele located near the cyclin-dependent kinase inhibitor 1A (*CDKN1A*) gene was associated with an increased proportion of slow-twitch muscle fibers in Russian (*n* = 151; *p* = 0.039), Finnish (*n* = 287; *p* = 0.03), and Japanese (*n* = 207; *p* = 0.008) cohorts (meta-analysis: *p* = 7.9 × 10^−5^. Furthermore, the frequency of the rs236448 A allele was significantly higher in Russian (*p* = 0.045) and Japanese (*p* = 0.038) elite endurance athletes compared to ethnically matched power athletes. On the contrary, the C allele was associated with a greater proportion of fast-twitch muscle fibers and a predisposition to power sports. *CDKN1A* participates in cell cycle regulation and is suppressed by the miR-208b, which has a prominent role in the activation of the slow myofiber gene program. Bioinformatic analysis revealed that the rs236448 C allele was associated with increased *CDKN1A* expression in whole blood (*p* = 8.5 × 10^−15^) and with greater appendicular lean mass (*p* = 1.2 × 10^−5^), whereas the A allele was associated with longer durations of exercise (*p* = 0.044) reported amongst the UK Biobank cohort. Furthermore, the expression of *CDKN1A* increased in response to strength (*p* < 0.0001) or sprint (*p* = 0.00035) training. Accordingly, we found that *CDKN1A* expression is significantly (*p* = 0.002) higher in the m. vastus lateralis of strength athletes compared to endurance athletes and is positively correlated with the percentage of fast-twitch muscle fibers (*p* = 0.018). In conclusion, our data suggest that the *CDKN1A* rs236448 SNP may be implicated in the determination of muscle fiber composition and may affect athletic performance.

## 1. Introduction

Human skeletal muscle consists of several types of muscle fibers, including type I (slow-twitch/oxidative), type IIA (fast oxidative), and type IIX (fast glycolytic) fibers, which exhibit different phenotypic properties. Type I muscle fibers have the lowest velocity of contraction and greatest resistance to fatigue, conversely, type IIX fibers exhibit the highest velocity of contraction and lowest resistance to fatigue [1]. 

In untrained individuals, the proportion of slow-twitch fibers in the vastus lateralis muscle may vary from 5 to 90% [2,3,4], which may affect their ability to perform aerobic or anaerobic exercise. For example, individuals with a greater proportion of slow-twitch muscle fibers can generally achieve a greater number of repetitions at 80% one-repetition maximum (1RM) during resistance training [5] and are more tolerant to long-distance exercise [6] compared to individuals that have a higher proportion of fast-twitch fibers. Accordingly, endurance athletes are reported to have a remarkably high proportion of type I fibers in their trained muscle groups [6,7,8], whereas the muscles of sprinters and weightlifters predominantly consist of IIA/IIX fibers [9].

Variability in the proportion of skeletal muscle fiber types may also explain marked differences in aspects of aging-associated diseases, including obesity, insulin resistance, and hypertension. For example, a low percentage of type I muscle fibers is a risk factor for the development of obesity, insulin resistance, and hypertension [6,10,11]. On the other hand, a high proportion of fast-twitch muscle fibers is associated with a low incidence of fractures in athletes [6].

The ratio of muscle fibers is determined by both environmental and genetic (heritable) factors. Adult skeletal muscle is highly malleable [12,13,14], and environmental factors such as training, sedentary behavior, and bed-rest may lead to a shift in the proportion of muscle fiber types. Nevertheless, a study of muscle fibers in monozygous and dizygous twins found the total contribution of heritable factors accounts for up to 45% of the variation in human muscle fiber composition [15]. These heritable factors may include DNA sequence variants, such as single nucleotide polymorphisms (SNPs), indels, and structural variations, which can alter gene expression and/or protein structure but, to date, relatively few candidates have been identified that specifically associate with fibre type. Genetic variants that are known to associate with differences in muscle fibre type, include angiotensin-converting enzyme (*ACE*) [16,17,18], alpha-actinin 3 (*ACTN3*) [18,19,20], type 2 angiotensin receptor (*AGTR2*) [21], brain derived neurotrophic factor (*BDNF*) [22], cerebellin 2 precursor (*CBLN2*) [23], copine 5 (*CPNE5*) [24], fat mass and obesity-associated (*FTO*) [25], hypoxia-induced factor 1-alpha (*HIF1A*) [26], immunoglobulin superfamily member 3 (*IGSF3*) [27], mitochondrial ORF of the 12S rRNA type-c (MOTS-c) encoded within the mitochondrial 12S rRNA [28], nuclear respiratory factor 1 (*NRF1*) [29], peroxisome-induced alpha receptor (*PPARA*) [30], peroxisome proliferator-activated receptor gamma coactivator 1-alpha (*PPARGC1A*) [29], striated muscle enriched protein kinase (*SPEG*) [31], transforming growth factor alpha (*TGFA*) [32], vascular endothelium growth factor receptor 2 (*VEGFR2*) [33] genes, with additional variants likely to exist. In addition, individual genetic variants may combine to influence the composition of muscle fibers through different pathways [34,35].

The aforementioned studies used a candidate gene design [36] to investigate genotype-phenotype associations between DNA polymorphisms and muscle fiber type. A limitation of this approach is that it cannot detect polymorphic variants that lie within non-coding (possibly, regulatory) regions of the genome. Genome-wide association studies (GWAS) using micro-array analysis represent a non-targeted method that has proved successful in uncovering new genetic associations to exercise-related phenotypes, especially when followed by replication studies in independent cohorts [37,38]. Therefore, the present study aimed to identify DNA polymorphisms associated with muscle fiber composition in three independent cohorts using a genome-wide approach, followed by two case-control studies involving athletes.

## 2. Materials and Methods

### 2.1. Ethical Approval

The Russian part of the study was approved by the Ethics Committee of the Federal Research and Clinical Center of Physical–Chemical Medicine of the Federal Medical and Biological Agency of Russia (protocol no. 2017/04). The Japanese part of the study was approved by the Ethics committee of the Juntendo University and Fukuoka University (Approval Code: GSHSS2021-2; Approval Date: 26 March 2021). The Finnish part of the study was approved by the Hospital District of Helsinki and Uusimaa (this data was used with permission; Database of Genotypes and Phenotypes (dbGaP) Study Accession: phs001048.v2.p1). Written informed consent was obtained from each participant. The study complied with the Declaration of Helsinki and ethical standards for sport and exercise science research.

### 2.2. Study Participants

#### 2.2.1. The Russian Cohorts

The Russian case-control study involved 219 elite athletes (110 men and 109 women), of which 69 were power athletes (100–400 m runners, 500–1000 m speed skaters, sprint cyclists, 50 m swimmers; mean age ± SD: 24.0 ± 3.0 years) and 150 elite endurance athletes (biathletes, 5–10 km speed skaters, cross-country skiers, 3–10 km runners, 0.8–25 km swimmers, race walkers, and triathletes; mean age ± SD: 24.2 ± 3.3 years). The athletes were Russian national team members (participants and prize winners in international competitions) who had never tested positive for doping. The Russian muscle biopsy study involved 151 physically active participants of Russian origin (101 men: mean age ± SD: 30.5 ± 8.0 years; mean height: 180.0 ± 6.2 cm; mean body mass: 80.6 ± 10.4 kg; mean percentage of fast-twitch muscle fibers: 52.9 ± 15.5%; mean percentage of slow-twitch muscle fibers: 50.1 ± 15.4%; cross-sectional area (CSA) of slow-twitch muscle fibers: 5417 ± 1236 μm^2^; CSA of fast-twitch muscle fibers: 6173 ± 2001 μm^2^; 50 women: mean age ± SD: 27.2 ± 7.4 years; mean height: 167.2 ± 5.3 cm; mean body mass: 59.3 ± 5.6 kg; mean percentage of fast-twitch muscle fibers: 48.7 ± 12.3%; mean percentage of slow-twitch muscle fibers: 54.3 ± 13.2%; CSA of slow-twitch muscle fibers: 4601 ± 1000 μm^2^; CSA of fast-twitch muscle fibers: 4273 ± 1397 μm^2^). The Russian gene expression study (analysis of *CDKN1A* gene expression in m. vastus lateralis) involved 7 strength (mean age ± SD: 32.6 ± 8.0 years; mean height: 178.4 ± 5.3 cm; mean body mass: 87.7 ± 14.2 kg) and 11 endurance (mean age ± SD: 33.3 ± 8.6 years; mean height: 182.4 ± 6.5 cm; mean body mass: 76.1 ± 10.5 kg) male athletes.

#### 2.2.2. The Japanese Cohorts

The Japanese case-control study involved 114 athletes (91 men and 23 women), of which 54 were elite power athletes (100–400 m runners, jumpers, and throwers; mean age ± SD: 28 ± 7 years) and 60 endurance runners (800 m to marathon; mean age ± SD: 24 ± 3 years). All of these athletes were international-level competitors. The Japanese muscle biopsy study involved 207 healthy individuals (101 men, age 46.7 ± 17.8 years; 106 women, age 48.2 ± 16.3 years).

#### 2.2.3. The Finnish Cohort

The Finnish muscle biopsy study involved 287 individuals (167 men, age 59.5 ± 8.1 years; 120 women, age 60.7 ± 7.4 years) from the FUSION study as previously described [39].

### 2.3. Evaluation of Muscle Fiber Composition by Immunohistochemistry

#### 2.3.1. Russian Study

Vastus lateralis samples were obtained from the left leg using the modified Bergström needle procedure with aspiration under local anesthesia using 2% lidocaine solution. Prior to analysis, samples were frozen in liquid nitrogen and stored at −80 °C. Serial cross-sections (7 μm) were obtained from frozen samples using an ultratom (Leica Microsystems, Wetzlar, Germany). Sections were thaw-mounted on Polysine glass slides, maintained at room temperature (RT) for 15 min and incubated in PBS (3 × 5 min). The sections were then incubated at RT in primary antibodies against slow or fast isoforms of the myosin heavy chains (M8421, 1:5000; M4276; 1:600, respectively; Sigma-Aldrich, Burlington, MA, USA) for 1 h and incubated in PBS (3 × 5 min). Afterwards, the sections were incubated at RT in secondary antibodies conjugated with FITC (F0257; 1:100; Sigma-Aldrich) for 1 h. The antibodies were removed, and the sections washed in PBS (3 × 5 min), placed in mounting media, and covered with a cover slip. Images were captured with a fluorescent microscope (Eclipse Ti-U, Nikon, Tokyo, Japan). All analyzed images contained 334 ± 14 fibers. The ratio of the number of stained fibers to the total fiber number was calculated. Fibers stained in serial sections with antibodies against slow and fast isoforms were considered hybrid fibers. The cross-sectional areas (CSAs) of fast- and slow-twitch muscle fibers were evaluated using ImageJ software (NIH, Bethesda, MD, USA).

#### 2.3.2. Japanese Study

Skeletal muscle samples were obtained from the vastus lateralis muscles of participants under sterile conditions and local anesthesia (1% lidocaine) using a disposable needle biopsy instrument (Max Core or Magnum; C. R. Bard, Covington, GA, USA). Biopsies were conducted under ultrasound imaging (Noblus; Aloka, Tokyo, Japan) to collect tissue samples from approximately 15 cm above the lateral epicondyle of both or either leg of each participant and avoided the inclusion of subcutaneous fat and the subfascial and myotendinous parts as far as possible. In addition, any visible non-muscle tissue (e.g., fat tissue) was removed from the biopsy samples. Then, the samples were immediately frozen in liquid nitrogen and stored at −80 °C until further analysis. Myosin heavy chain (MHC) protein isoforms (I, IIa, and IIx) were analyzed by the use of sodium dodecyl-sulfate polyacrylamide gel electrophoresis (SDS-PAGE) as markers of muscle fiber composition, according to a previously described method [40].

#### 2.3.3. Finnish Study

Muscle fiber composition in 287 Finnish individuals was estimated based on the expression of the myosin heavy chain 1 (MYH1), myosin heavy chain 2 (MYH2), myosin heavy chain 7 (MYH7), Ca^2+^ ATPase A1 and Ca^2+^ ATPase A2 genes, as previously described [39]. Muscle samples were obtained from the vastus lateralis using a conchotome, under local anesthesia, with 20 mg∙ml^−1^ lidocaine hydrochloride without epinephrine.

### 2.4. Genotyping

#### 2.4.1. Russian Study

Molecular genetic analysis was performed with DNA samples obtained from leukocytes. Four milliliters of venous blood were collected in tubes containing EDTA (Vacuette EDTA tubes; Greiner Bio-One, Kremsmünster, Austria). DNA was extracted on the same day. DNA extraction and purification were performed using a Technosorb commercial kit (Technoclon, Moscow, Russia) according to the manufacturer’s instructions. The genotyping process was performed using HumanOmni1-Quad BeadChips or HumanOmniExpress BeadChips (Illumina, San Diego, CA, USA) to genotype > 900,000 SNPs. The assay required 200 ng of the DNA sample as input with a concentration of at least 50 ng/µL. Exact concentrations of the DNA in each sample were measured using a Qubit Fluorometer (Invitrogen, Waltham, MA, USA). All further procedures were performed according to the instructions of the Infinium High-Density Assay.

#### 2.4.2. Japanese Study

Total DNA was isolated from saliva or venous blood using the Oragene DNA Collection Kit (DNA Genotek, Ontario, Canada) or the QIAamp DNA blood Maxi Kit (QIAGEN, Hilden, Germany), respectively. The total DNA content was measured using the NanoDrop 8000 spectrophotometer (Thermo Fisher Scientific, Waltham, MA, USA). Subsequently, DNA samples were adjusted to a concentration of 50 ng/μL with Tris-EDTA buffer and stored at 4 °C. Total DNA samples were genotyped using the HumanOmniExpress Beadchip (Illumina, San Diego, CA, USA) or Japonica SNP array [41] to genotype > 700,000 SNPs, according to the manufacturer’s instructions. Genotype calls were performed with Illumina GenomeStudio software and PLINK 1.9 (National Institutes of Health, Bethesda, MD, USA) was used for quality control checks.

#### 2.4.3. Finnish Study

DNA samples were extracted from the blood and the polymorphisms were genotyped using the HumanOmni2.5–4v1_H BeadChip array (Illumina, San Diego, CA, USA), as previously described [39].

### 2.5. Gene Expression Analysis

#### 2.5.1. Total RNA Isolation

Prior to the muscle biopsy of vastus lateralis of the left leg (in the morning), athletes were asked not to train for one day to analyze their gene expression profiles at the resting state. RNeasy Mini Fibrous Tissue Kit (Qiagen, Hilden, Germany) was used to isolate RNA from 18 muscle tissue samples of Russian athletes (7 strength and 11 endurance athletes). Frozen tissue samples were placed in a box submerged in liquid nitrogen. Each sample was transferred without thawing on a sterile Petri dish placed on a frozen plastic ice pack. A piece of tissue with a weight of 10 mg was separated with a sterile scalpel and immediately placed in a 2 mL safe-lock microcentrifuge tube containing 300 µL of lysis buffer and one sterile stainless steel bead with a diameter of 4 mm. Samples were homogenized using the TissueLyser II system (Qiagen, Hilden, Germany) with shaking twice for 2 min at 25 Hz. RNA samples were isolated according to the manufacturer’s guidelines. RNA concentration was measured using the Qubit spectrophotometer (Thermo Fisher Scientific, Waltham, MA, USA). RNA quality was assessed using the BioAnalyzer electrophoresis system and BioAnalyzer RNA Nano assay (Agilent Technologies, Santa Clara, CA, USA). RNA integrity number (RIN) was calculated for each RNA sample. Only RNA samples with RIN > 7 were included in the study. Samples were stored at −80 °C until sequencing libraries were prepared.

#### 2.5.2. RNA Sequencing

Total RNA samples were treated with DNAse I using Turbo DNA-free Kit (Thermo Fisher Scientific, Waltham, MA, USA) according to the kit guidelines. Libraries for RNA sequencing were prepared using NEBNext Ultra II Directional RNA Library Prep Kit for Illumina with the NEBNext rRNA Depletion Module (New England Biolabs, Ipswich, MA, USA). RNA libraries were sequenced on the HiSeq system (Illumina, San Diego, CA, USA) with 250 cycles. Sequenced reads were pseudoaligned to hg38 gencode (v37) transcriptome using kallisto v0.48.0 [42] with default settings. Gene level expression abundances were calculated using the tximport Bioconductor package [43]. Expression of the *CDKN1A* gene was presented in transcripts per kilobase million (TPM).

### 2.6. Statistical Analyses

Statistical analyses were conducted using GraphPad InStat Version 3.05 (GraphPad Software, Inc., San Diego, CA, USA) software. PLINK 1.9 program (National Institutes of Health, Bethesda, MD, USA) was used to perform genetic data quality control and PLINK 2.0 was used to perform principal component analysis and association testing via generalized linear models [44]. Bcftools was used for vcf files conversion. Phasing and imputation of genotypes were done using shapeit2 and impute2 programs. The chi-square test (χ^2^) was used to test for the presence of the Hardy–Weinberg equilibrium (HWE). Thereafter, the frequencies of genotypes or alleles were compared between power and endurance athletes using Fisher’s exact test. The mean differences between groups were compared using an unpaired *t*-test. To perform the meta-analysis with obtained data, the Cochrane Review Manager (RevMan) (London, UK) version 5.3 was used. A random effect model was applied. The odds ratio with 95% confidence intervals (CI) was estimated using the Mantel–Haenszel method. The heterogeneity degree between the studies was assessed with the I² statistics. All data are presented as mean (SD). The *p*-Values < 0.05 were considered statistically significant.

## 3. Results

### 3.1. Muscle Fiber Composition and Athlete Status Studies

**Figure 1:**
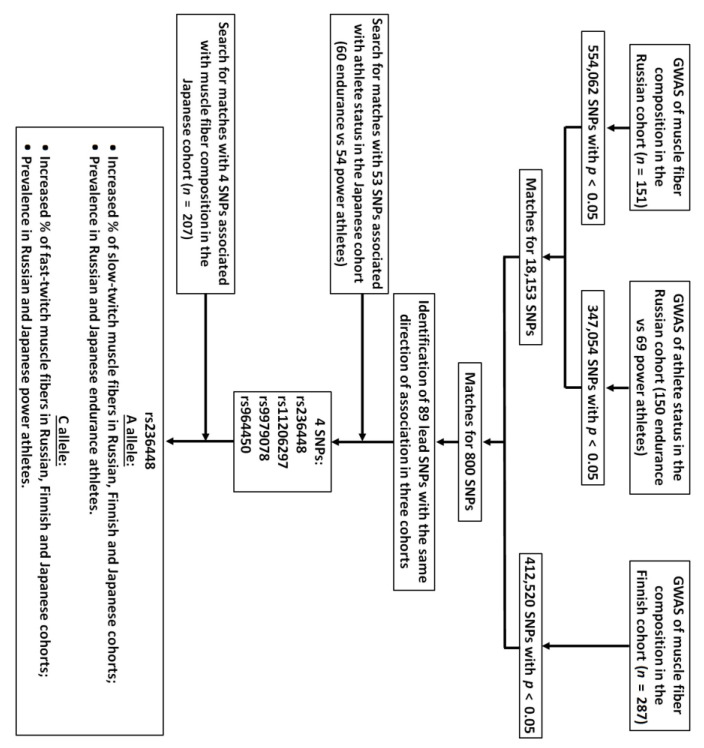
A schematic overview of the study design and findings.

A flow diagram displaying the study design and findings is shown in Figure 1. In the first stage, we performed two genome-wide association studies (GWASes) using Russian cohorts only.

The first GWAS was performed using muscle fiber composition data of 151 participants (adjusted for sex, age, level, and type of physical activity). Although none of the SNPs reached genome-wide significance level (i.e., *p* < 5 × 10^−8^), we felt justified to use all nominally associated (*n* = 554,062; *p* < 0.05) SNPs in the following steps to prevent the loss of potentially important findings. The second GWAS involved 150 endurance and 69 power athletes from Russia. In this case-control study we have identified 347,054 SNPs nominally associated with athlete status (with no genome-wide significant associations). By combining two datasets, we found matches for 18,153 common SNPs.

In the second stage, we performed a GWAS of muscle fiber composition data of 287 participants from Finland (adjusted for sex and age) and found 412,520 nominally (*p* < 0.05) associated SNPs (with no genome-wide significant associations). By combining two datasets from Russian (18,153 SNPs) and Finnish (412,520 SNPs) cohorts, we found matches for 800 common SNPs. Most of these SNPs were in linkage disequilibrium (LD) with each other, and some SNPs were in the opposite direction of association when different cohorts were compared. Out of 800 SNPs, we selected 89 lead (independent and non-linked) SNPs with the same direction of association in three cohorts (for example, the same allele should be associated with an increased proportion of slow-twitch muscle fibers in Russian and Finnish cohorts and over-represented in Russian endurance compared to power athletes) (Supplementary Table S1).

In the third (replication) stage, we used genomic data from two Japanese cohorts. Of 89 SNPs discovered in the second stage, 53 were available for the replication study in Japanese cohorts of athletes (i.e., endurance vs. power athletes). Of 53 SNPs, four alleles have been shown to be over-represented in Japanese endurance compared to power athletes (rs236448 A (*p* = 0.038; odds ratio (OR) = 2.1), rs9979078 A (*p* = 0.033; OR = 1.8), rs11206297 C (*p* = 0.047; OR = 2.4), rs964450 G (*p* = 0.029; OR = 2.3)). Finally, we have checked if these four SNPs were associated with muscle fiber composition in the Japanese cohort (*n* = 207). Only one SNP rs236448 A/C located in the intergenic region (6p21.2) has been shown to be associated with muscle fiber composition (adjusted for sex and age) and athlete status in Japanese cohorts, as well as in all tested cohorts. More specifically, the rs236448 A was associated with an increased proportion of slow-twitch muscle fibers in the Russian (*n* = 151; *p*= 0.039, β = 3.46), Finnish (*n* = 287; *p* = 0.03, β = 2.78) and Japanese (*n* = 207; *p* = 0.008, β = 12.11) cohorts (meta-analysis: *p* = 7.9 × 10^−5^) (Supplementary Table S2). This also indicates that the opposite allele (rs236448 C) was associated with an increased proportion of fast-twitch muscle fibers (*p* < 0.05). The rs236448 genotype explained 2.4%, 1.8%, and 1.9% of the variation in the percentage of slow-twitch muscle fibers in Russian, Finnish and Japanese subjects, respectively.

The genotypic frequencies of the rs236448 SNP for both endurance and power athletes in Russian and Japanese cohorts were in Hardy–Weinberg equilibrium. The frequency of the rs236448 A allele was significantly higher in Russian (77.3 vs. 68.1%; *p* = 0.045; OR = 1.6) and Japanese (87.5 vs. 76.9%; *p* = 0.038; OR = 2.1) elite endurance athletes compared to ethnically matched power athletes (Table 1).

The pooled OR favoring endurance athletes compared with power athletes was 1.89 (95% CI 1.18–3.01, *p* = 0.008 for the random effect model of meta-analysis) for the rs236448 AA genotype. No heterogeneity between studies (I^2^ = 0%; *p* = 0.32) was observed. Overall, we found that the AA genotype was associated with a predisposition to endurance sports, while the carriage of the C allele (rs236448 AC or CC genotypes) was associated with the ability to become a power athlete in both ethnicities.

### 3.2. Bioinformatic and Gene Expression Studies

Bioinformatic and literature analysis revealed that the rs236448 is located near the cyclin-dependent kinase inhibitor 1A (*CDKN1A*) gene, which participates in cell cycle regulation. First, we hypothesized that the discovered rs236448 A/C polymorphism might be functional and alter *CDKN1A* gene expression. Indeed, according to the eQTLGen database, the rs236448 C allele has been reported to be associated with increased *CDKN1A* expression in the whole blood (*p* = 8.5 × 10^−15^) in a large cohort of subjects (*n* = 31,684) [45].

Next, we hypothesized that the rs236448 C allele (the predictor of an increased proportion of fast-twitch muscle fibers) would be associated with power-related traits and the A allele (predictor of an increased proportion of slow-twitch muscle fibers) with endurance-related traits in the UK Biobank cohort. Accordingly, it has been reported that the C allele was associated with greater appendicular lean mass (*p* = 1.2 × 10^−5^) in 450,243 subjects [46], while the A allele with longer duration of exercises (*p* = 0.044) in 172,650 individuals [47].

**Figure 2:**
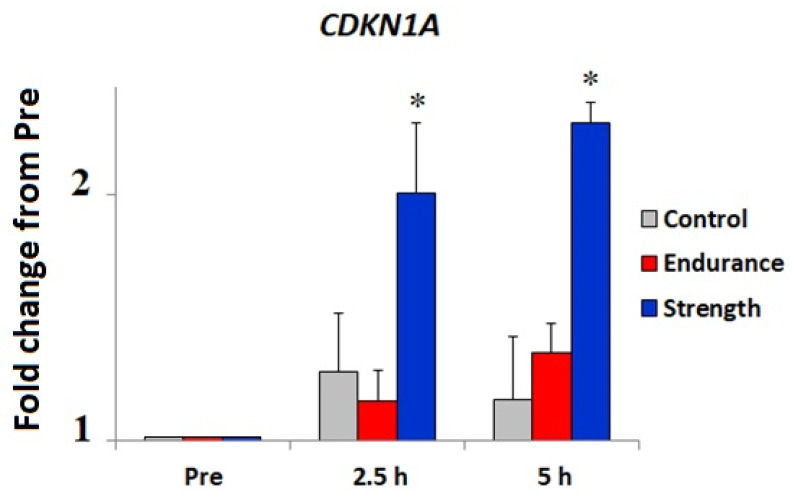
Effect of acute endurance (*n* = 7) and strength (*n* = 7) exercise on *CDKN1A* gene expression of m. vastus lateralis of young men. Time points corresponding to Pre, 2.5, and 5 h post-exercise are shown. * *p* < 0.0001. This figure was generated using publicly available data from Vissing and Schjerling [48].

**Figure 3:**
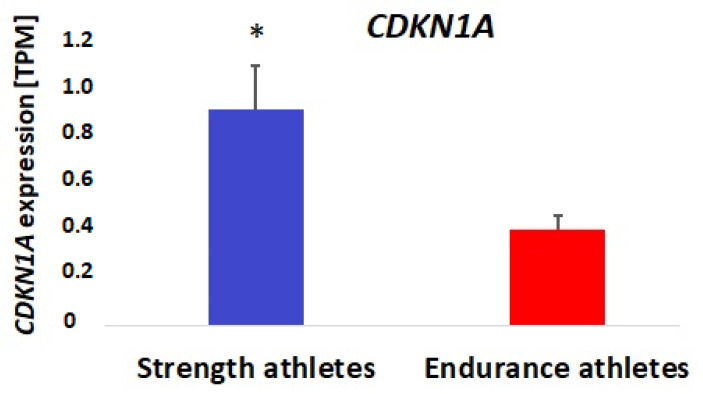
Differences in the resting *CDKN1A* gene expression between strength (*n* = 7) and endurance (*n* = 11) athletes. * *p* = 0.002.

Finally, using the available human skeletal muscle transcriptome dataset [48], we have checked the effects of a single-bout endurance (*n* = 7) and resistance (*n* = 7) exercise on the mRNA of the *CDKN1A* gene. It has been reported that the expression of the *CDKN1A* gene of m. vastus lateralis of young men significantly increases (by 2–2.4 fold) in response to strength training (*p* < 0.0001) (Figure 2). Accordingly, using our own transcriptomic data, we found that *CDKN1A* gene expression was significantly higher (0.91 (0.18) vs. 0.40 (0.04) TPM; *p* = 0.002) in the m. vastus lateralis of strength athletes (*n* = 7) compared to endurance athletes (*n* = 11) (Figure 3). Furthermore, *CDKN1A* gene expression was positively correlated with the percentage of fast-twitch muscle fibers in this cohort (r = 0.55, *p* = 0.018).

## 4. Discussion

To the best of our knowledge, this is the first GWAS of muscle fiber composition. Since power and endurance, as well as fast- and slow-twitch muscle fibers are located at opposite extremes of the muscle performance continuum and are highly correlated with each other, we used a multi-stage genome-wide approach with five independent cohorts (three datasets of muscle fiber composition and two datasets of athlete status). This resulted in the identification of a highly replicable genetic marker *CDKN1A* rs236448 A/C for muscle fiber composition and predisposition to sports. Specifically, the rs236448 A allele is associated with an increased proportion of slow-twitch muscle fibers, endurance athlete status, and physical performance (long duration of exercise), while the C allele is associated with a higher percentage of fast-twitch fibers, predisposition to power sports, and greater lean mass.

*CDKN1A* encodes a cyclin-dependent kinase inhibitor 1A (also known as p21) which is involved in cell cycle regulation (including stem cell proliferation), transcription, apoptosis, DNA repair, and cell motility [49]. These functions are performed through the ability of p21 to interact with a number of proteins involved in these processes [50]. Recently, *CDKN1A* was predicted and then validated as a target gene of miR-208b [51]. MiR-208b overexpression significantly reduces the expression of *CDKN1A* mRNA and protein by binding to its 3’-UTR [51]. MiR-208b plays a dominant role in the specification of muscle fiber identity by activating slow- and repressing fast-myofiber gene programs [52] and may activate AMPK/PGC-1a signaling and mitochondrial biogenesis [53]. One might suggest that MiR-208b, by binding to the *CDKN1A,* may influence muscle cell proliferation and differentiation.

Using an available transcriptome dataset [48], we identified that the gene expression of *CDKN1A* in m. vastus lateralis of young men significantly increases in response to strength training. Similarly, acute sprint exercise may also increase *CDKN1A* gene expression (*p* = 0.00035) of vastus lateralis in males (*n* = 7) [54,55]. Both strength and sprint training are considered predominantly anaerobic types of exercise, which require the recruitment of fast-twitch muscle fibers. Accordingly, we also found that *CDKN1A* gene expression was significantly higher in the m. vastus lateralis of strength athletes compared to endurance athletes and was positively correlated with the percentage of fast-twitch muscle fibers. This data indicates that the CDKN1A may be an important factor involved in the adaptation to strength/sprint training.

Bioinformatic analysis revealed that the rs236448 polymorphism might be functional, with the C allele being associated with higher *CDKN1A* gene expression in whole blood in a large cohort of subjects [45]. Furthermore, the C allele has been reported to be associated with greater appendicular lean mass in the UK Biobank cohort [46], which is in line with the positive role of the *CDKN1A* gene in the adaptation to strength/sprint training and the association of C allele with an increased proportion of fast-twitch muscle fibers. On the other hand, the A allele has been shown to be associated with longer duration of exercises in the UK Biobank cohort [47], which is consistent with the fact that the A allele is related to the increased proportion of slow-twitch (fatigue-resistant) muscle fibers.

**Figure 4:**
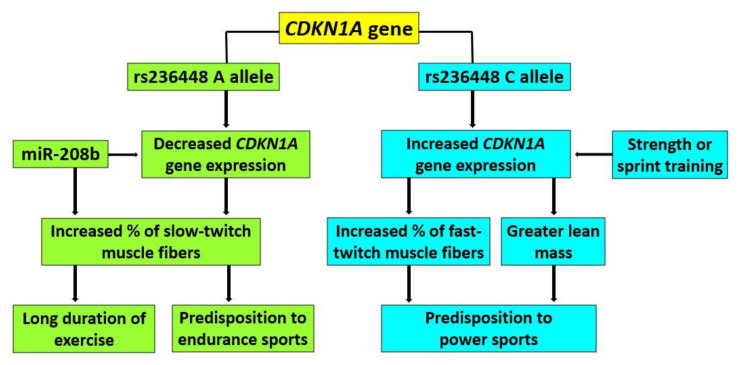
Scheme showing the hypothesized mechanism for the association of rs236448 alleles and various factors with muscle fiber composition and exercise-related phenotypes.

Overall, Figure 4 shows the hypothesized mechanism for the association of different *CDKN1A* rs236448 alleles and various factors with muscle fiber composition and exercise-related phenotypes (based on experimental, literature and bioinformatic data). However, mechanisms through which altered expression of the *CDKN1A* gene influences muscle fiber composition, lean mass, and athletic performance remain speculative, and further in vitro and in vivo studies of gene function are required. Interestingly, *CDKN1A* rs236448 (location: 6p21.2) is in a very weak LD with *CPNE5* gene rs3213537 (location: 6p21.2; D’: 0.1903; r^2^: 0.0035), and homeostatic iron regulator (*HFE*) gene rs1799945 (location: 6p22.2; D’: 0.1223; r^2^: 0.0018), and each of these polymorphisms were previously linked with muscle fiber composition [24,35]. This indicates that variants located at chromosome 6p21-6p22 represent one of the most prominent areas associated with muscle fiber composition. Further studies are needed to detect lead (and likely causal) SNPs located at 6p21-6p22.

Our study does have limitations. Our combined sample size of subjects with muscle fiber data was not large enough (*n* = 645) to detect loci at a genome-wide significant level (i.e., *p* < 5 × 10^−8^). To overcome this issue, we have used a multi-stage approach, including different study designs (i.e., genotype-phenotype and case-control genetic studies, as well as gene expression studies) and different closely related phenotypes in several ethnicities (Finnish, Japanese, Russian, and British). We also performed a meta-analysis of muscle fiber data, which enabled us to increase statistical power. However, an observed statistical association between a genetic marker and a phenotype does not necessarily mean a causal relationship, and further mechanistic studies are warranted to investigate the possible mechanisms related to the *CDKN1A* rs236448 SNP. For example, transgenic and knockout animal models are required to test the hypothesis that *Cdkn1a* overexpression increases the proportion of fast-twitch muscle fibers, while the *Cdkn1a* deletion promotes a fast-to-slow transition. Our muscle fiber composition study included individuals of a wide age range (Russians: 29.4 ± 7.9, Japanese: 47.5 ± 17.1, Finnish: 60.0 ± 7.8 years) and with different types and levels of physical activity, which could affect the results. However, we have adjusted our results for age and other covariates to minimize such differences. The frequency of the CC genotype in Japanese subjects was relatively low, and a larger sample size may be needed to detect the effect of this genotype on muscle fiber composition in East Asians. Finally, it should be noted that muscle fiber composition varies across muscles in humans (for example, there may be differences between gastrocnemius, deltoid, vastus lateralis, and soleus of the same individual). Therefore, the link between the *CDKN1A* rs236448 SNP and muscle fiber types should be explored in other muscle groups as well.

## 5. Conclusions

In conclusion, using a multi-stage genome-wide approach, we have identified that the rs236448 A allele is associated with an increased proportion of slow-twitch muscle fibers, physical performance, and endurance athlete status. On the contrary, the C allele is associated with an increased proportion of fast-twitch muscle fibers, greater muscle mass, and a predisposition to power sports. Our data suggest that the *CDKN1A* rs236448 SNP may be implicated in the determination of muscle fiber composition and may affect athletic performance. Nevertheless, given the substantial heritable component of muscle fiber composition, there are clearly more—and probably many more—genetic variants associated with muscle fiber composition that remain to be identified.

## Figures and Tables

**Table 1:** The rs236448 SNP genotypes distribution and allelic frequencies in Japanese and Russian cohorts of athletes.

Group	*n*	rs236448 Genotypes			A Allele, %	*p* Value (OR) (Endurance vs. Power)
		AA	AC	CC		
Japanese endurance athletes	60	46	13	1	87.5	0.038 * (2.1)
Japanese power athletes	54	30	23	1	76.9	
Russian endurance athletes	150	89	54	7	77.3	0.045 * (1.6)
Russian power athletes	69	33	28	8	68.1	

* *p* < 0.05 (Fisher’s exact test), statistically significant differences between ethnically matched endurance and power athletes. OR, odds ratio.

## Data Availability

RNA-seq data reported in this paper have been deposited in GEO with accession number GSE200398.

## Supplementary Materials

The following supporting information can be downloaded at: https://www.mdpi.com/article/10.3390/cells11233910/s1, Table S1: Most significant SNPs associated with muscle fiber composition and athlete status in different cohorts. Table S2: The percentage of slow-twitch muscle fibers in subjects with different rs236448 genotypes across three ethnicities.
